# The Fate of Activated Group 2 Innate Lymphoid Cells

**DOI:** 10.3389/fimmu.2021.671966

**Published:** 2021-04-22

**Authors:** Laura Mathä, Itziar Martinez-Gonzalez, Catherine A. Steer, Fumio Takei

**Affiliations:** ^1^ Terry Fox Laboratory, British Columbia Cancer, Vancouver, BC, Canada; ^2^ Department of Microbiology, Tumor and Cell Biology, Karolinska Institutet, Solna, Sweden; ^3^ Department of Pathology and Laboratory Medicine, University of British Columbia, Vancouver, BC, Canada

**Keywords:** innate lymphoid cells, type 2 inflammation, mucosal immunity, immunological memory, neonatal immunity, exhaustion, transdifferentiation, migration

## Abstract

Group 2 innate lymphoid cells (ILC2s) reside in both mucosal and non-mucosal tissues and play critical roles in the first line of defense against parasites and irritants such as allergens. Upon activation by cytokines released from epithelial and stromal cells during tissue damage or stimulation, ILC2s produce copious amounts of IL-5 and IL-13, leading to type 2 inflammation. Over the past 10 years, ILC2 involvement in a variety of human diseases has been unveiled. However, questions remain as to the fate of ILC2s after activation and how that might impact their role in chronic inflammatory diseases such as asthma and fibrosis. Here, we review studies that have revealed novel properties of post-activation ILC2s including the generation of immunological memory, exhausted-like phenotype, transdifferentiation and activation-induced migration.

## Introduction

Group 2 innate lymphoid cells (ILC2s) belong to the family of innate lymphoid cells that include natural killer (NK) cells, helper innate lymphoid cells (ILCs) and lymphoid tissue inducer (LTi) cells, which all share a common lineage. Helper ILCs are classified into three groups, based on their similarities to helper T cell subtypes with respect to the expression of transcription factors and effector cytokines: ILC1s are T-bet^+^ and produce interferon (IFN) γ, ILC2s highly express GATA3 and release IL-5 and IL-13, while ILC3s are dependent on RAR-related orphan receptor (ROR) γt and secrete IL-22 and IL-17 (1). ILC2s were first identified in 2010 as a population of innate immune cells with a lymphoid morphology which lacks the expression of lineage markers commonly expressed by T, B and myeloid cells (2–4). They were initially termed natural helper cells (2), nuocytes (3) or innate helper type 2 (Ih2) cells (4), but unification of their nomenclature was proposed in 2013, and are now called ILC2s (5). Since their initial discovery in the gut, they have been identified in various other organs in mice, including the lung (6–8), skin (9–11), adipose tissues (12–14), liver (15), pancreas (16) and heart (17, 18), as well as in humans (6, 9, 10, 13, 19) and their pathologic and protective roles in multiple human diseases such as asthma, atopic dermatitis and fibrosis, and infections have been described. ILC2s also play a critical role in adipose tissue homeostasis by sustaining type 2 immunity and promoting beiging of white adipose tissue (12–14), while an ILC2-tuft cell circuit orchestrates intestinal homeostasis and remodeling (20, 21).

ILC2s are known to seed tissues early during development and adapt to environmental cues (22–27); consequently, their phenotype slightly differs depending on their residing organs (**Table 1**). MouseILC2s express CD45, IL-7Rα (CD127), CD90 (Thy1) and IL-2Rα (CD25) (2–4), while CD103 (11) and IL-18Rα (22) are uniquely expressed by skin ILC2s. In contrast, the expression of IL-33 receptor (ST2) and IL-25 receptor varies depending on the organ. ST2 is expressed by the lung (6, 8), liver (15) and adipose tissue ILC2s (12–14), while ILC2s in the small intestine (SI) express low levels of this receptor (28) and the expression levels vary in the skin (9, 22, 28). In contrast, IL-25R is highly expressed by SI ILC2s (20), while adipose tissue ILC2s do not express IL-25R (22) and both IL-25R positive and low/negative ILC2s have been reported in the skin (11, 20, 22). Interestingly, we have previously shown that the majority of naïve lung ILC2s are negative for this marker, whereas its expression is induced by activation (29).

**Table 1:** Summary of ILC2 phenotypes.

	Mouse					Human
	Lung	Intestine	Skin	Liver	Fat	Blood
**CD127**	+	+	+	+	+	+
**CD90**	+	+	+	+	+	−
**CD25**	+	+	+	+	+	+
**CD103**	ND	ND	+	ND	ND	−
**IL-18Rα**	−	−	+	−	−	+
**ST2**	+	low	+/low	+	+	−
**IL-25R**	low	+	+/low	ND	−	−
CRTH2*						+
CD161*						+
**GATA3**	+	+	+	+	+	+

“+”, expressed; “−”, not expressed; “low”, expressed at low levels; “+/low”, various reports exist; “ND”, not determined. *human ILC2 markers. Human ILC2 phenotype is based on peripheral blood ILC2s.

ILC2s in human peripheral blood (PB) are similar to mouse tissue ILC2s in that they express CD127 and CD25, but are uniquely identified by the expression of chemoattractant receptor-homologous molecule expressed on Th2 cells (CRTH2) and CD161 (19). Unlike mouse ILC2s, human PB ILC2s lack the expression of CD90, ST2, IL-25R and CD103 (19, 30, 31), whereas they are positive for IL-18Rα expression (31).

Due to the species and tissue differences in surface molecule expression and the lack of universal markers for ILC2s, identification of ILC2s can be challenging. Additionally, some surface molecules are downregulated or upregulated upon ILC2 activation or in disease conditions, making it more difficult to definitively identify them. However, the transcription factor GATA3 is highly expressed by mouse and human ILC2s, and therefore, it is a useful marker for ILC2 identification (32, 33).

## Activation of ILC2s

ILC2s lack antigen specific receptors and hence, their activation is finely regulated by a repertoire of molecules including cytokines, neurotransmitters and lipid mediators. Here, we review the activation of ILC2s by cytokines (direct) and allergens (indirect). The regulation of ILC2 activation and inhibition by other molecules is reviewed in great detail elsewhere (34, 35). Upon activation, ILC2s produce IL-5 and IL-13 among others, resulting in type 2 inflammation characterized by eosinophilia, alternative activation of macrophages, type 2 helper T (Th2) cell differentiation and IgE class switching.

### Direct Activation by Cytokines

Alarmins are molecules that are normally present within cells and released upon tissue injury, environmental insults, physiological stress or necrosis (36). They induce activation of various immune cells, resulting in sterile inflammation. ILC2s express receptors for an alarmin cytokine, IL-33, and other secreted cytokines such as IL-25, thymic stromal lymphopoietin (TSLP) and IL-18, and are potently activated by these cytokines.

IL-33 is constitutively expressed in the nuclei of endothelial cells, epithelial cells, fibroblastic reticular cells and adventitial stromal cells (37–42), while IL-25 can be either constitutively expressed as seen in tuft cells (21), or its expression can be induced in immune cells, such as alveolar macrophages (43), mast cells (44), basophils, eosinophils (45) and Th2 cells (46). TSLP expression is induced in epithelial cells in the lung, such as alveolar type II cells (42), whereas it is constitutively expressed in the large intestine (47, 48). Stimulation of IL-33 and IL-25 signaling pathways through their cognate receptors consisting of ST2 and IL-1 receptor accessory protein (IL-1RAcP) (49), and IL-17RA and IL-17RB (50), respectively, induces activation of nuclear factor (NF) κB and mitogen-activated protein kinase (MAPK) (50, 51). This leads to phosphorylation of GATA3, promoting its binding to *Il5* and *Il13* promoters and ILC2 proliferation (52). TSLP binds to the receptor comprised of IL-7Rα and TSLPR (53) and activates a separate downstream signaling pathway involving Janus kinase (JAK) 1/2 and STAT5 (54). STAT5 binds to the *Gata3* gene and together, they regulate ST2 expression, enhancing IL-33 induced activation (55).

In vivo responsiveness of ILC2s towards cytokine stimulation varies depending on their residing tissues. Intranasal (i.n.) administration of recombinant IL-33 potently activates lung ILC2s (29, 56, 57). In contrast, naïve adult lung ILC2s do not respond to i.n. IL-25 or TSLP administration (29, 42, 56, 58, 59). Interestingly, lung ILC2s upregulate IL-25R as they acquire memory-like properties after allergen or IL-33 treatment and become responsive to IL-25 stimulation (29). We have also shown that naïve neonatal lung ILC2s are potently stimulated by IL-25 (27). Unlike lung ILC2s, ILC2s in mesenteric lymph nodes (mLN), spleen and liver expands upon intraperitoneal (i.p.) administration of IL-25 (3, 4). Moreover, IL-25 deficient mice have significantly reduced ILC2s in the SI at steady state and after worm infection, suggesting a critical role of IL-25 in maintenance and activation of intestinal ILC2s (21). IL-25 also serves as the predominant cytokine for skin ILC2 activation in BALB/c mice (10).

Despite their potency in activating ILC2s *in vivo*, IL-33 or IL-25 alone is not sufficient to induce ILC2 activation *in vitro*, suggesting that activation of ILC2s requires a secondary signal provided by co-stimulatory cytokines such as IL-2 and IL-7 (2, 7, 10).

It was recently shown that the majority of mouse skin ILC2s express IL-18Rα (22). Consequently, ILC2s isolated from skin are weakly activated upon *in vitro* stimulation by IL-18 + TSLP compared to TSLP only, which does not activate them (22). ILC2 stimulation through this pathway is physiologically significant, as IL-18 deficient mice have impaired skin ILC2 activation and type 2 inflammation in a mouse model of atopic dermatitis-like disease (22). Of note, IL-18 mediated activation of ILC2 is independent of IL-33, IL-25 and TSLP (22). Human PB ILC2s, unlike mouse ILC2s, respond very potently to *in vitro* IL-18 + IL-7 stimulation by producing type 2 cytokines (31), suggesting that IL-18 may play a more essential role in human ILC2 biology.

### Indirect Activation by Allergens

ILC2s play a crucial role in airway allergic diseases induced by various allergens in experimental mouse models. Due to the lack of antigen specific receptors, ILC2s do not directly recognize and respond to allergens. Instead, they are stimulated by various cytokines released upon irritation or tissue injury caused by allergen inhalation.

In murine models, various allergens including house dust mite (HDM) (56, 57, 60, 61), chitin (62), papain (29, 63), fungal allergens from *Alternaria* (64–66) and *Aspergillus* (29) species and ovalbumin (OVA) (56, 58) have been shown to activate lung ILC2s. Treatment with these allergens causes disruption of the barrier integrity and induces the release of ILC2-activating cytokines from the airway epithelial cells (7, 42, 59, 64, 66–69). As *Crlf2* (TSLPR), *Il25* and *Il1rl1* (ST2) triple-deficient mice have similar numbers of ILC2s as wild-type mice upon chitin treatment (62), and *Il33* ^-/-^ mice have normal ILC2 accumulation in bronchoalveolar lavage fluid (BALF) after HDM administration (70), these cytokines may not be necessary for ILC2 proliferation upon allergen stimulation. However, they appear to play a critical role in ILC2 activation as *Il33* ^-/-^ or *Il1rl1* ^-/-^ mice have reduced ILC2 cytokine production compared to wild-type mice in papain, *Alternaria* and HDM models (63, 64, 70), and mice deficient in one or more ILC2-activating cytokine signaling pathways show significantly reduced expansion of eosinophils and alternatively activated macrophages (AAM) after chitin treatment (62).

Interestingly, HDM driven ILC2 expansion requires T cell activation, as mice with impaired T cell activation do not mount ILC2-mediated type 2 inflammation upon HDM challenge (70), while adaptive lymphocytes are not necessary in the papain model, as i.n. papain treatment into *Rag1* ^-/-^ mice leads to normal ILC2 activation and eosinophilia (7). In contrast, ILC2s are not required for allergic inflammation in an OVA model, in which Th2 cells are stimulated by OVA plus adjuvant alum, as ILC2 deficient mice show no impairment of type 2 immune responses (61). These data suggest that immunological environment induced by these allergens seems to vary and the role of ILC2s in type 2 inflammation may be slightly different in each model.

## Function of ILC2s

Upon activation, lung ILC2s secrete copious amounts of type 2 cytokines, IL-5 and IL-13 but also IL-9 and amphiregulin (6, 7, 42, 71, 72). IL-5 induces eosinophil development and recruitment, resulting in eosinophilia in the lung (73). IL-13 causes goblet cell hyperplasia, followed by mucus hyperproduction, smooth muscle contraction and subepithelial fibrosis, resulting in tissue remodeling and airway hyperresponsiveness (74). Moreover, together with IL-4, IL-13 provides cues for macrophage differentiation into AAM (74). ILC2-derived IL-13 has been shown to disrupt tight junctions in the epithelial lining of the lung causing leakiness of the airways (75). It also facilitates differentiation of helper T cells into Th2 type by inducing migration of activated dendritic cells to the draining LN (63), further enhancing type 2 inflammation. In addition to type 2 cytokines, ILC2s transiently produce IL-9 early during inflammation (42, 71, 72), which provides signals for mast cell accumulation and airway remodeling by inducing goblet cell hyperplasia (76, 77). IL-9 also promotes survival (72) and/or activation (42) of ILC2s in an autocrine/paracrine manner (42, 71), resulting in amplification of IL-5 and IL-13 production.

More recent studies have shown that IL-10 producing ILC2s, termed “ILC2_10_” and “exhausted ILC2s” (discussed in more detail later) are generated in the mouse lung during chronic inflammation or in response to IL-2 (78, 79). The release of IL-10 can also be induced by mouse lung and SI ILC2s *in vitro* by various stimulants, including IL-2, IL-10, IL-27, IL-4, retinoic acid (RA) or neuromedin U (78, 80). Interestingly, blocking IL-10 results in a reduction in IL-10 production from ILC2s, suggesting an autocrine/paracrine regulation by IL-10 (80). In humans, a KLRG1^+^ subset of ILC2s secrete IL-10 when stimulated in the presence of RA (81, 82). While GATA3 expression confirms their ILC2 lineage identity, IL-10^+^ ILC2s express CTLA4 and CD25, resembling regulatory T cells (81, 82). Consequently, they inhibit various CD4^+^ helper T cell subsets and ILC2s in an IL-10 dependent manner (81, 82), suggesting previously unappreciated immune regulatory role of ILC2s.

In addition to soluble mediators like cytokines, ILC2s directly interact with other ILC2s and lymphocytes through a repertoire of surface molecules. ILC2s have been shown to directly facilitate Th2 polarization through PD-1/PD-L1 interaction during *Nippostrongylus brasiliensis* infection (83), while IL-33 activated ILC2s stimulate Th2 and regulatory T cells *via* OX40/OX40L interaction (84). Activated ILC2s, expressing ICOS ligand (ICOSL), also promote accumulation of ICOS^high^ regulatory T cells through ICOS/ICOSL interactions (85). Interestingly, ILC2s express both ICOS and ICOSL and provide survival signals to each other (86).

Due to ILC2’s potent capacity to produce cytokines, they initiate robust type 2 immune responses upon epithelial injury or environmental insults, and consequently, they are implicated in pathogenesis of various airway diseases, including asthma (87, 88), chronic rhinosinusitis with nasal polyps (CRSwNP) (19, 89–93), allergic rhinitis (94) and pulmonary fibrosis (95–97), and respiratory infections caused by influenza (8) and respiratory syncytial (98, 99) viruses. In contrast, ILC2s play a crucial protective role during influenza virus infections by producing amphiregulin, which facilitates the repair of damaged airway epithelium and restores the impaired lung function caused by influenza (6). Moreover, human IL-10^+^ ILC2s prevent the loss of epithelial barrier integrity upon allergen exposure (81), suggesting a protective role in airway allergic diseases. Overall, the role of ILC2s in type 2 inflammation seem to vary in different models and diseases.

## The Fate of Activated ILC2s

**Figure 1:**
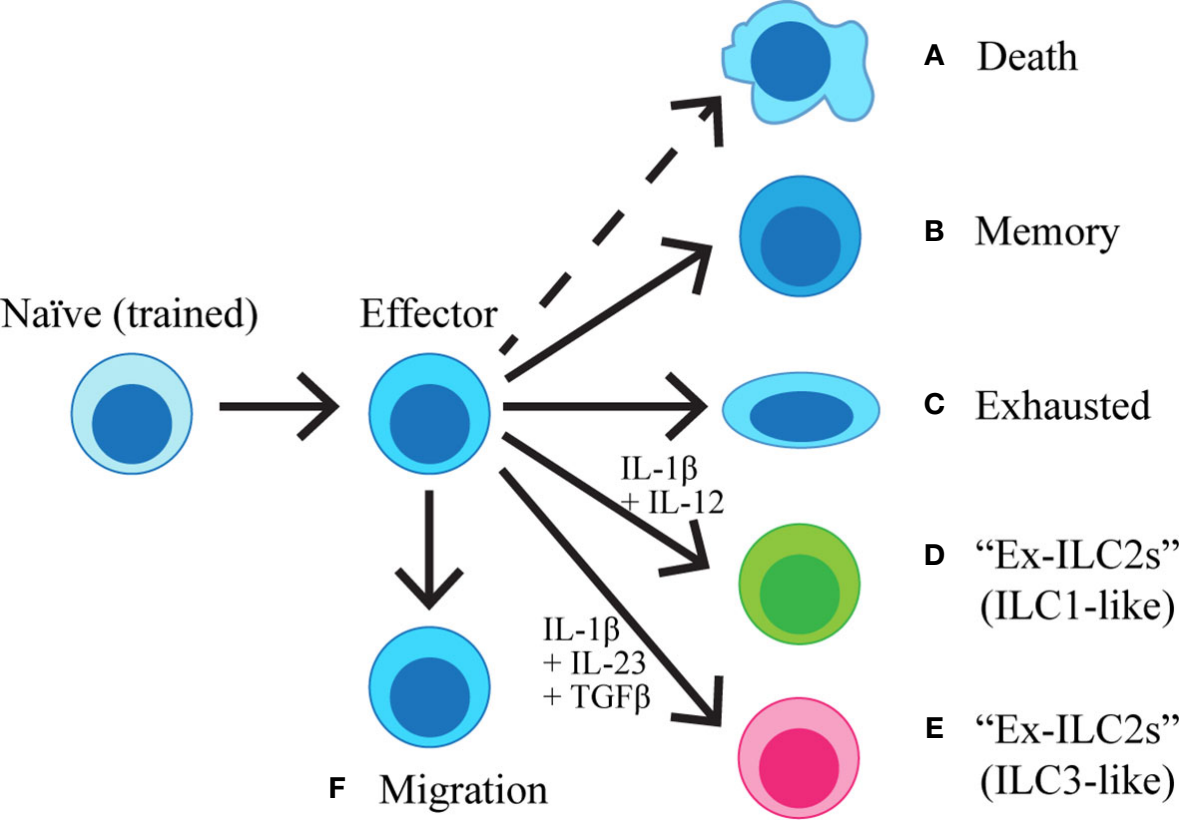
Naïve ILC2s are exposed to IL-33 during the neonatal period and become “trained”. Once activated, ILC2s become effector cells, producing cytokines and initiating the inflammatory cascade. The majority of the effector cells are predicted to die **(A)**, while a proportion of ILC2s acquires immunological memory **(B)**. Some ILC2s may become exhausted **(C)**, whereas others can also transdifferentiate into IFNγ-producing ILC1-like cells **(D)** or IL-17-producing ILC3-like cells **(E)** upon stimulation with IL-1β + IL-12 or IL-1β + IL-23 + TGFβ, respectively. A subset of ILC2s migrates out of the lung and enter circulation **(F)**.

Once activated and having proliferated, lung ILC2s propagate a series of inflammatory events by production of type 2 cytokines as described above. The majority of activated lung ILC2s remain in the lung (23, 100, 101) and presumably die once their task is completed, although activation-induced death of ILC2s has not yet been studied. A small proportion of activated lung ILC2s persists in the lung as memory-like ILC2s (29), or becomes exhausted (79). Some ILC2s may transdifferentiate into other types of ILCs under specific conditions (102–106), whereas it was recently found that a subset of ILC2s leaves the residing organs and circulates (101, 107) (**Figure 1**, **Table 2**).

**Table 2:** The fate of activated ILC2s.

	Models (mouse/human)	References
Immunological memory/trained immunity	Mouse	(27, 29, 108)
Exhaustion	Mouse	(79)
Transdifferentiation to ILC1s	Human	(102–104)
	Mouse	(103)
Transdifferentiation to ILC3s	Human	(105, 106)
Activation-induced migration	Mouse	(101, 107, 109)

### ILC2 Memory

Immunological memory is the ability of immune cells to recall a previous encounter with a specific antigen and mount robust responses upon subsequent exposures to the same antigen. The acquisition of immunological memory is a hallmark of adaptive immunity and innate leukocytes have long been thought to not possess the ability to remember previous activation. However, with the discovery of memory NK cells (110–113), the potential of innate lymphocytes to acquire memory-like properties have been unraveled. Detailed reviews of immunological memory of ILC2s and innate leukocytes can be found elsewhere (114, 115).

#### Memory ILC2s in Mouse

We have recently shown that lung ILC2s can acquire immunological memory (29). Upon allergen inhalation, mouse lung epithelium releases alarmins such as IL-33, which activates lung resident ILC2s. This results in a vigorous expansion of ILC2s, followed by a contraction phase and resolution of lung inflammation as indicated by the decrease in lung eosinophils and BALF type 2 cytokines. While the number of ILC2s continues to decrease after the peak of inflammation, some of the activated ILC2s survive for a long time as shown by persistence of ILC2s labelled with bromodeoxyuridine (BrdU), which was administered at the time of initial activation (29). These ILC2s are more responsive to a secondary stimulus compared to naïve ILC2s, which is a feature of immunological memory, and hence, were termed “memory-like ILC2s” (29). However, unlike antigen specific T and B cells, ILC2s do not recognize specific antigens and consequently, memory-like ILC2s are able to mount robust immune responses to subsequent stimulation by unrelated allergens or cytokines. Memory-like ILC2s are defined by changes in the expression of a small subset of genes compared to naïve or effector ILC2s (29). These changes include the upregulation of *Il17rb*, a subunit of the IL-25 receptor complex, which enables them to uniquely react to *in vivo* IL-25 stimulation, unlike naïve lung ILC2s. Although adoptive transfer of memory-like ILC2s and gene expression data indicate that the acquisition of immunological memory is cell-intrinsic (29), we cannot rule out the possibility that environmental components may be required to mount an efficient recall response.

A similar memory-like ILC2 population has been demonstrated in mouse models of nematode infections. Yasuda et al. found that ILC2s persist in BALF for at least 1 month after infection of mice with the migratory helminth *Strongyloides venezuelensis*, while other immune cell numbers go back to similar levels as in naïve mice (108). Upon challenge with the unrelated nematode *N*. *brasiliensis* a month later, mice pre-infected with *S*. *venezuelensis* demonstrate protective effects against *N*. *brasiliensis* infection, accompanied by increased number of ILC2s and cytokines in the BALF of pre-infected mice compared to those without primary infection. The protective effect is not specific to *S. venezuelensis*, as similar results are obtained when the order of infection is switched (108). In contrast, IL-33 pre-treatment fails to induce a similar protection (108). The resistance against *N. brasiliensis* infection is independent of CD4^+^ T cells, suggesting a primary role of ILC2s, whereas IL-33 and eosinophils are critical. Unlike the memory-like ILC2s we have previously described (29), the enhanced responsiveness of ILC2s upon challenge infection by nematodes is not mediated by IL-25R upregulation and hence, the authors named these cells “trained ILC2s (108).”

Seehus and colleagues have defined a new subset of IL-10 producing ILC2s in mouse lungs, termed “ILC2_10_” (78). These cells are generated upon i.p. administration of high dose IL-33 or chronic stimulation with i.n. papain treatment and act as regulatory cells with an immunosuppressive function. Interestingly, although ILC2_10_ contracts quickly upon withdrawal of stimulation, administration of a single dose of IL-33 a month after the initial stimulation induces an increased number of ILC2_10_ in pre-treated mice compared to untreated mice (78). Therefore, it is likely that immunological memory can be acquired by different subsets of ILC2s and this may have implications not only in the exacerbation but also in regulation of lung inflammation.

It is important to note that *in vitro* or *in vivo* primed Th2 cells can also demonstrate innate-like properties by responding to cytokines or unrelated antigens (116). Such innate-like behavior of Th2 cells is TCR independent and requires IL-33, suggesting that it is an antigen non-specific response (116). Th2 cells in mice inoculated with *N. brasiliensis* can mount an efficient airway eosinophilic inflammation upon HDM challenge 3-4 weeks later, suggesting their resemblance to the innate immunological memory of ILC2s (116). Therefore, immunological memory should be defined as the capacity of immune cells to remember previous activation and respond more efficiently upon reactivation despite antigen specificity (114).

#### Memory ILC2s in Human

Although immunological memory-like properties have been described in human NK cells (117, 118), there is little evidence of memory ILC2s in human. A recent paper by van der Ploeg identified the human counterpart of the inflammatory ILC2s (iILC2s) (119), which is a subtype of ILC2s induced during inflammation (discussed in more detail later) (109). These human iILC2s, characterised by the expression of CD45R isoform CD45RO, are enriched in the blood and nasal polyps of patients with CRSwNP and in the blood of asthma patients, and are highly activated. CD45RO^+^ ILC2s differentiate from resting CD45RA^+^ ILC2s isolated from PB upon *in vitro* cytokine stimulation. While CD45RO is a marker for human memory T cells (120), whether or not some of these highly activated iILC2s are able to retain the expression of CD45RO and become memory ILC2s remains to be explored.

#### Trained ILC2s (Neonatal ILC2s)

In vivo lineage-tracing experiments have revealed that a portion of steady-state ILC2s in various mouse tissues originates in the neonatal period and persists into adulthood (23, 27). ILC2s are undetectable in the lung of newborn mice, but ILC2 numbers rapidly increase in the following 10 days and peak on postnatal days 10-14 when the numbers reach to ~2-3 times of those in adult mouse lungs (24–26). In parallel to the ILC2 expansion, the level of IL-33 expression is significantly higher in neonatal lungs than adult lungs (24, 26, 27). It is thought that endogenous IL-33 is released from stromal cells in the neonatal period (41) due to postnatal mechanical stress from the first newborn breaths (26), development (25), or hyperoxia (24, 121). At the peak of their expansion, neonatal ILC2s upregulate activation-related genes such as *Mki67, Il13, Il5*, and *Il1r2*, increase intracellular expression of IL-13 and IL-5, and expression of Ki67 and IL-25R (24, 27). Neonatal lungs also have ILC2-dependent eosinophilia (24, 26, 27). In IL-33 deficient pups, ILC2s develop normally but they do not expand as in wild-type pups and their activation is not observed (24, 26). Administration of recombinant sST2, a decoy receptor that blocks IL-33 signaling, also inhibits the activation of neonatal lung ILC2s (25). These studies together showed that neonatal lung ILC2s are activated by endogenous IL-33.

While the neonatal lung ILC2s contract to adult levels around 3 weeks of age, ILC2s labeled by i.n. BrdU administration during the expansion phase persist into adulthood (27). Schneider et al. also irreversibly marked neonatal ILC2s by tamoxifen-induced Cre expression and red fluorescent protein (RFP) and showed that RFP-marked neonatal ILC2s persist for many months and are only very slowly replaced by RFP-negative ILC2s in adulthood (23). Therefore, ILC2s that develop in the neonatal period become tissue resident cells in adulthood. Interestingly, i.n. injections of IL-33 into adult mice previously given BrdU in the neonatal period more intensely activate BrdU-positive than negative lung ILC2s (27). Lung ILC2s in adult IL-33 knock out (KO) mice do not respond as intensely to i.n. IL-33 stimulation as wild-type ILC2s, and i.n. administration of IL-33 in the neonatal period reverses the impaired response of ILC2s in IL-33KO mice in adulthood (27). Ricardo-Gonzalez et al. also showed that absence of IL-33 signaling leads to downregulation of adult ILC2 type 2 signatures (22). These studies suggested that activation of lung ILC2s by endogenous IL-33 in the neonatal period has long lasting effects on ILC2 functions in adulthood and the effect was termed “ILC2 training (27).” The idea of trained ILC2s implies that lung resident ILC2s in adult mice are more responsive to stimuli than ILC2s that develop in adulthood as the former are trained in the neonatal period whereas the latter are untrained, similar to ILC2s in IL-33KO mice.

It should be noted that there are significant differences between memory-like ILC2s and neonatally trained ILC2s. Neonatal ILC2s transiently upregulate IL-25R but the levels diminish into adulthood and adult lung ILC2s do not respond to i.n. IL-25 stimulation (27). In contrast, memory-like ILC2s maintain IL-25R expression and are activated by i.n. IL-25 treatment (29).

### Exhausted ILC2s

Exhaustion of lymphocytes occurs upon persistent antigen stimulation, where immunological memory generation fails and cells are also unable to execute effector functions (122). Miyamoto et al. reported that ILC2s lacking core binding factor (Cbf) β showed an activated phenotype at steady state but a hyporesponsive phenotype upon *in vitro* stimulation (79). The gene expression profiles of Cbfβ deficient ILC2s stimulated by IL-33 *in vitro* resemble those of exhausted CD8^+^ T cells, and hence, these cells were termed “exhausted-like ILC2s”. A similar population of exhausted-like ILC2s, defined by their expression of IL-10 and TIGIT, are generated in a severe subacute asthma model, where wild-type mice are treated with high doses of papain short-term (79). These ILC2s, which express killer cell lectin-like receptor G1 (KLRG1), programmed cell death protein 1 (PD-1) and glucocorticoid-induced TNFR-related (GITR), appear in the BALF, where the inflammation is the most severe, but not in the lung. They do not proliferate well nor do they produce much type 2 cytokines. The exhausted-like ILC2s are also generated in the lung as well as in the BALF when wild-type mice receive a high-dose papain treatment every 3 days over a month period (79). The exhausted phenotype of ILC2s is further enhanced by Cbfβ deficiency, suggesting that the ILC2 exhaustion process is inhibited by Runx/Cbfβ complex. It is important to note that exhausted-like ILC2s are different from previously-described ILC2_10_ (78), as the former lacks the ability to efficiently produce type 2 cytokines, whereas the latter is a great producer of type 2 cytokines.

### Ex-ILC2s

Plasticity of ILC2s towards IFNγ producing ILC1s (102–104) and IL-17 producing ILC3s (105, 106) have been documented. An indication of ILC2’s capacity to transdifferentiate into ILC1s came from the observation that PB and lung samples collected from chronic obstructive pulmonary disease (COPD) patients are enriched in ILC1s whereas the frequency of ILC2s is reduced compared to healthy subjects or less severe COPD patients (102, 103). In vitro stimulation of ILC2s isolated from PB of healthy donors in the presence of IL-2, IL-1β (or IL-33 + TSLP) and IL-12 causes the loss of ILC2 signatures, such as GATA3, IL-5 and IL-13, and upregulation of ILC1 features, T-bet and IFNγ expression (102, 104). Priming of ILC2s with IL-1β is necessary, during which IL-1β epigenetically modifies the transcriptome of ILC2s, shifting it to a more ILC1-like profile (104). Interestingly, the ILC2 phenotype is restored when ex-ILC2s are stimulated with IL-4, whereas true ILC1s do not covert to ILC2s upon IL-4 stimulation (102). A similar transdifferentiation of ILC2s to ILC1s have been shown in mice using a model of influenza infection, where immunodeficient mice adoptively transferred with GFP^+^ ILC2s were infected with influenza virus (103). GFP^+^ ILC2s underwent downregulation of GATA3 and upregulation of IL-18Rα and IL-12Rβ2, and produced IFNγ upon ex vivo stimulation with IL-12 and IL-18 (103).

Human ILC2s have also been shown to have the capacity to convert into IL-17 producing Ckit^+^ NKp44^-^ ILC3-like cells when cultured in the presence of IL-2 + IL-1β + IL-23 + TGFβ (105, 106). This transdifferentiation is associated with downregulation of GATA3, followed by upregulation of RORγt expression and IL-17 production (105). IL-1β is required for IL-17 production, while TGFβ further promotes transdifferentiation by enhancing IL-17 production and RORγt expression, and inhibiting IL-5 production (105, 106). ILC2 phenotype and function are partially restored in IL-17^+^ ILC2s upon IL-4 stimulation. A marked increase in NKp44^-^ ILC3s is observed in psoriatic lesions and nasal polyps collected from cystic fibrosis patients (105, 106), suggesting that ILC2 transdifferentiation into ILC3s is physiologically relevant in the presence of the appropriate cytokine milieu.

Notably, the majority of studies demonstrating ILC2 plasticity have been performed using human samples. It is most likely due to the fact that human ILCs are constantly exposed to various stimuli and hence, their identities as different subsets of ILCs are more ambiguous than in mice, which are maintained relatively sterile in animal facilities. Therefore, it is more feasible to induce transdifferentiation of human ILC2s into ILC1s or ILC3s upon stimulation with type 1 or type 3 skewing cytokines, respectively, compared to mouse ILC2s.

### Migration of ILC2s

The majority of mouse lung ILC2s are generated during the neonatal period and there is a limited amount of *de novo* generation of ILC2s during adulthood (23). The idea of ILC tissue residency was first proposed by Gasteiger and colleagues, where they examined the composition of ILC pools at homeostasis and during inflammation using a parabiosis model (100). At steady state, more than 95% of ILCs are host-derived in all tissues analyzed, including the SI, salivary gland, lung and the liver (100). Upon infection with the migratory helminth, *N. brasiliensis*, the composition of donor and host-derived ILC2s in the lung, SI and mLN remains unchanged during the early stage of helminth infection, indicating that ILC2s are tissue resident lymphocytes unlike circulatory T and B cells.

**Figure 2:**
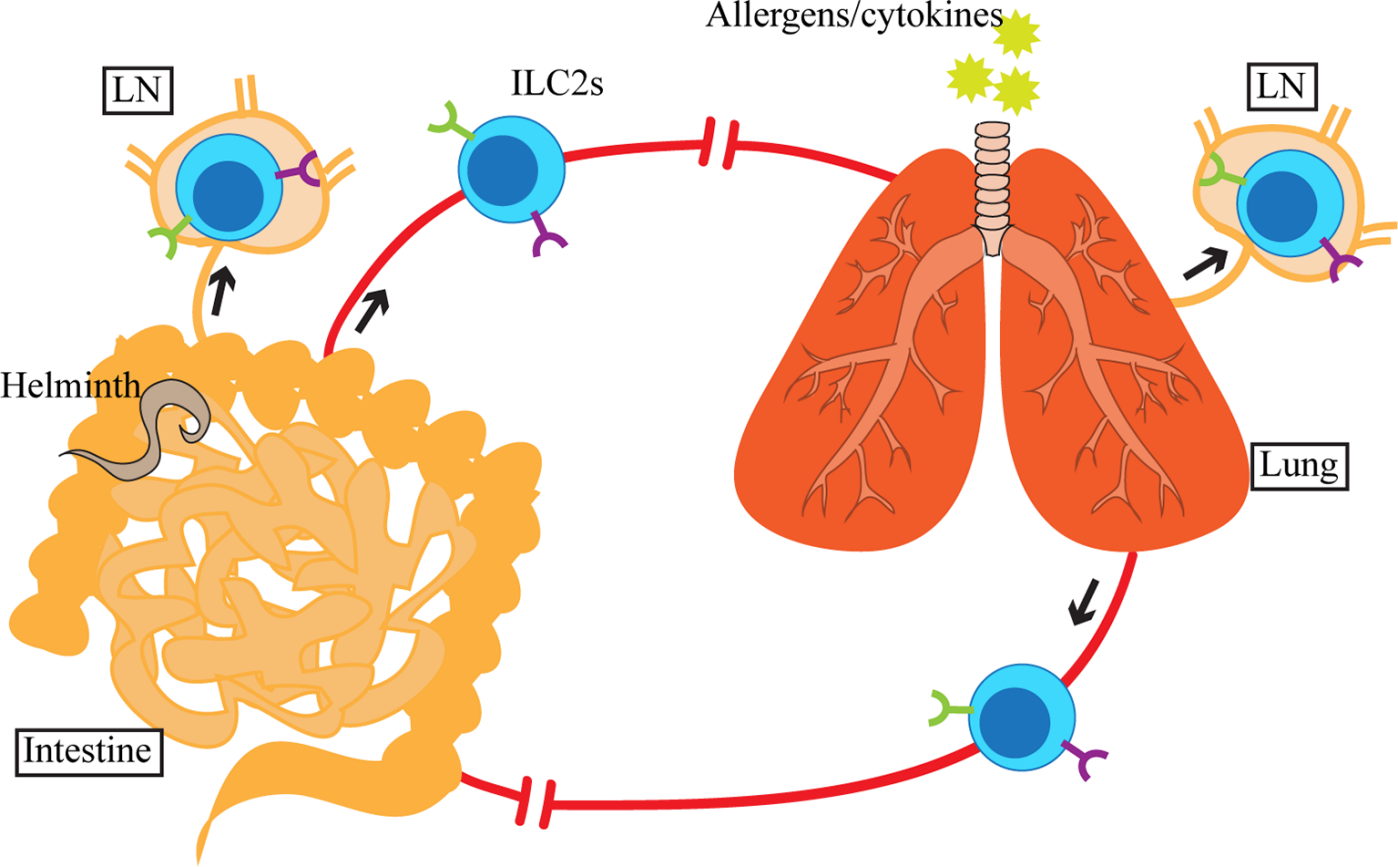
Upon activation by allergens, cytokines or helminths in the lung or small intestine (SI), ILC2s and inflammatory ILC2s (iILC2s) appear in circulation as well as in the tissue-draining lymph nodes (LN). SI-derived iILC2s migrate to the lung, whereas the destination of activated SI and lung ILC2s is currently unclear. Although ILC2s and iILC2s are detected in blood and LN, which suggests that their migration involves lymphatics and blood, the exact route of migration has yet to be determined.

While tissue residency of ILC2s is a well-established concept in the mouse system, in humans we can extrapolate from the presence of elevated ILC2s in asthmatic patients’ PB (88) that ILC2s may have the capacity to circulate or migrate. In mice, despite limited influx of hematogenous sources of ILC2s at homeostasis or during inflammation, i.p. IL-25 treatment or *N. brasiliensis* infection causes circulation of iILC2s, an inflammation-induced population of ILC2s identified as Lin^-^KLRG1^+^ST2^lo^ cells (107, 109). iILC2s originate in the SI, from which they enter the lymphatic and blood circulation before reaching the peripheral organs. We have also previously shown that activation of ILC2s in the lung by i.n. IL-33 or papain treatment causes accumulation of ILC2s in the draining mediastinal LN (29). Although we did not specifically investigate their migration from the lung to the LN, these data together with iILC2 migration indicate that migratory behavior of ILC2s may be induced upon activation (**Figure 2**).

A recent report by Ricardo-Gonzalez et al. examined the source of circulating ILC2s upon *N. brasiliensis* infection using fate-mapping models (101). Upon subcutaneous administration, *N. brasiliensis* first migrates to the lung, where it is coughed up and swallowed, entering the digestive tract, reaching the intestine (123). Early during the parasite infection (day 5), the majority of circulating ILC2s are dependent on IL-25 signaling and are of intestinal origin. In contrast, lung ILC2s, dependent on IL-33 signaling appear in circulation later during infection (day 12). These results demonstrate that in spite of their tissue residency at homeostasis and during inflammation, local activation of ILC2s can cause systemic dissemination of type 2 inflammation by inducing migratory behavior of ILC2s. It remains to be determined whether the circulating ILC2s return to their residing organs or migrate to other tissues.

## Concluding Remarks

The past few years of research on ILC2s at mucosal and non-mucosal sites has provided a wealth of knowledge regarding the regulation and functions of tissue resident ILC2s in various inflammatory conditions. In contrast, our understanding of what happens to ILC2s after activation and resolution of inflammation is still limited. While the majority of activated ILC2s are expected to die as the ILC2 population contracts after the peak of inflammation, some may become memory-like ILC2s, exhausted ILC2s, ex-ILC2s, or circulatory ILC2s, as summarized in this review. Considering that humans are continuously exposed to extrinsic insults, our ILC2s are presumably activated at some point. Therefore, we will likely benefit from future research on the dynamics of ILC2s after activation in various organs and the functional significance of previously activated ILC2s in inflammation at local and distant sites. It remains to be elucidated how and why activated tissue resident ILC2s take different pathways, namely death, memory, exhaustion, transdifferentiation or migration and whether it is predetermined by neonatal training or decided during activation. Finding specific markers for individual ILC2 populations will help us understand the processes.

## Author Contributions

LM wrote and edited the manuscript and generated the figures and tables. IM-G and CS wrote and edited the manuscript. FT reviewed the drafts, provided critical input and edited the manuscript and figures. All authors contributed to the article and approved the submitted version.

## Funding

This work was supported by a grant from the Canadian Institute of Health Research (PJT-153304).

## Conflict of Interest

The authors declare that the research was conducted in the absence of any commercial or financial relationships that could be construed as a potential conflict of interest.
